# Association Between Direct Oral Anticoagulants and Wunderlich Syndrome: A Case Report and a Comprehensive Analysis of the Literature and Evidence

**DOI:** 10.7759/cureus.91433

**Published:** 2025-09-01

**Authors:** Ayaz Khan, Yu-Hsiang Andy Chuang, Agapios Gkentzis, Neil Harvey

**Affiliations:** 1 Department of Urology, Royal Bolton Hospital, Bolton, GBR

**Keywords:** anticoagulant complications, direct oral anticoagulant, edoxaban, ruptured subcapsular haematoma, spontaneous renal haemorrhage, urological emergency, wunderlich syndrome

## Abstract

Wunderlich syndrome (WS) is a rare condition, referring to spontaneous retroperitoneal haemorrhage, often linked to renal neoplasms and, infrequently, to anticoagulant use. We describe a case of a 79-year-old male with spontaneous renal bleeding whilst on edoxaban, in the absence of other risk factors. The patient presented with ambiguous symptoms, prompting an initial misdiagnosis of a urinary tract infection. Subsequent imaging demonstrated a right renal subcapsular haematoma with active bleeding, managed by selective arterial embolisation. The patient subsequently developed a persistent, infected collection, requiring multiple drainages and repeat embolisation due to concerns of ongoing haemorrhage. Multidisciplinary care led to a gradual resolution, and anticoagulation was withheld, with close follow-up. Our case highlights diagnostic challenges in anticoagulated patients with non-specific symptoms and underscores the importance of early imaging, careful anticoagulation management, and thorough embolisation. Given the limited data on WS risk across anticoagulants, further research and standardised management protocols are needed.

## Introduction

Wunderlich syndrome (WS) is a rare clinical condition that refers to spontaneous, atraumatic renal haemorrhage into the subcapsular, perirenal and/or pararenal spaces [1]. Most patients complain of non-specific symptoms, although a minority may present with Lenk’s triad, consisting of flank pain, flank mass, and hypovolaemic shock. WS is commonly associated with renal neoplasms (e.g. angiomyolipomas and renal cell carcinomas), which account for roughly 60% of all cases, whereas vascular pathology (e.g. aneurysms and vasculitis) accounts for approximately 30%. The remaining 10% of cases arise due to more uncommon factors such as infections, cystic lesions, and calculi [1].

In the modern era, increased anticoagulation usage has led to a growing number of reported cases linking such agents to WS. In anticoagulated patients, the haemostatic balance is disrupted, increasing the risk of spontaneous bleeding [2]. Our report aims to review and analyse the current literature on the pathophysiology, clinical presentation, and management of WS in the context of anticoagulant use, underscoring the importance of early recognition and prompt intervention.

## Case presentation

A 79-year-old male presented to the Emergency Department with a three-day history of general malaise, visible haematuria, right flank pain, and lower urinary tract symptoms, including frequency and dysuria. His past medical history consisted of mitral regurgitation, atrial fibrillation (AF) managed with a permanent pacemaker, rheumatoid arthritis, chronic kidney disease (stage II), and osteoarthritis. He regularly took edoxaban, methotrexate, lansoprazole, enalapril, and folic acid. He was fully independent in activities of daily living and mobilised freely. On clinical examination, right renal angle tenderness was elicited. Observations revealed a heart rate of 97 beats per minute and a blood pressure of 119/60 mmHg. Initial laboratory investigations revealed: urea 10.1 mmol/L, creatinine 146 µmol/L (baseline 87 µmol/L), C-reactive protein (CRP) 243.8 mg/L, white cell count (WCC) 14.4 x10⁹/L, and haemoglobin (Hb) of 117 g/L. The clinical picture was initially suggestive of an upper urinary tract infection (UTI), and the patient was discharged on oral cefalexin with no planned follow-up.

**Figure 1:**
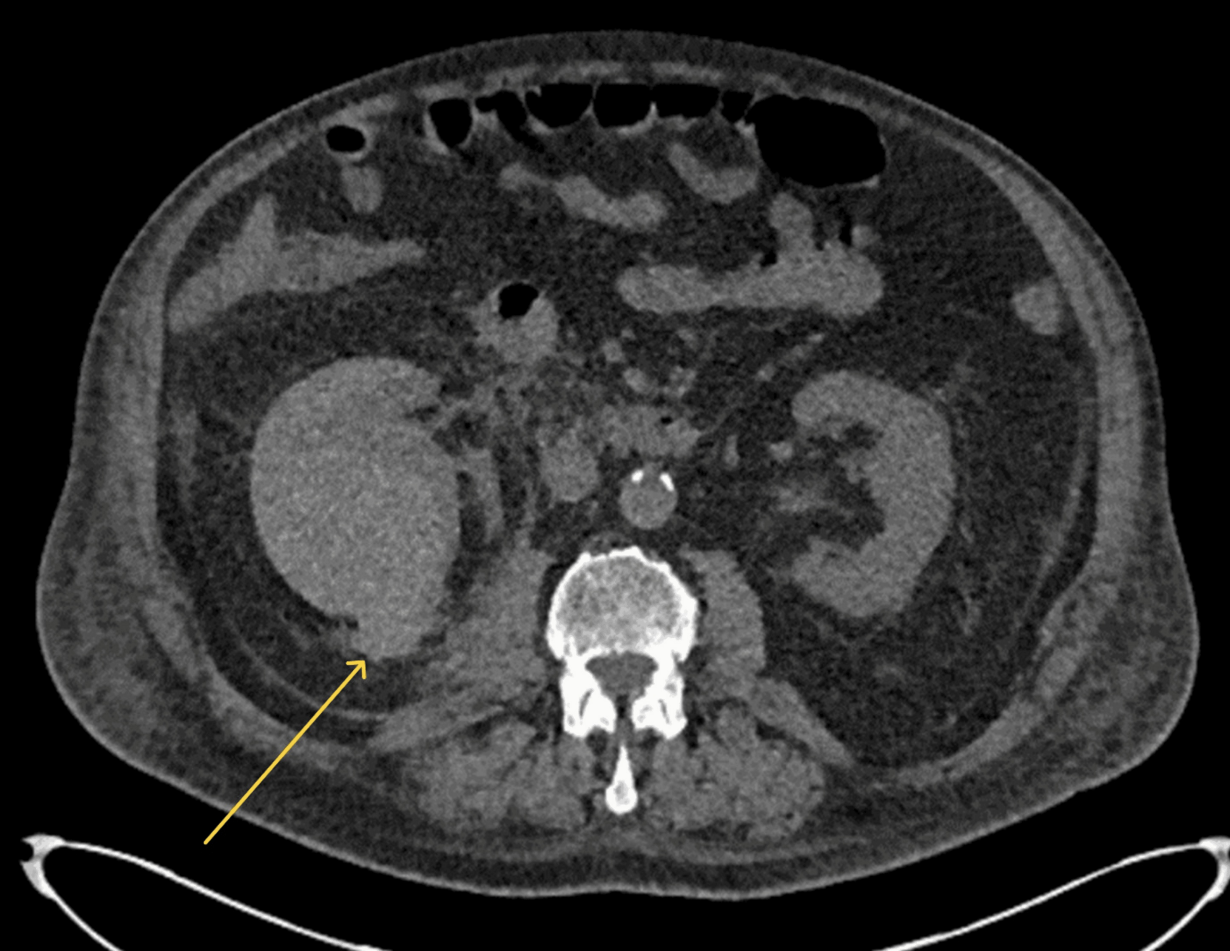
CT abdomen (axial view) in the portal venous phase The right kidney is oedematous with hyperdense peripheral content (arrow), suggesting a subcapsular haematoma. The hyperdensity tracks posteriorly to the perinephric space, indicating cortical disruption. CT, computed tomography

However, the following day, he re-presented with worsening right flank pain and new-onset dyspnoea. A chest X-ray revealed a moderately enlarged heart and chronic right basal changes, but, given persistent pyrexia and raised inflammatory markers, the patient was admitted with a working diagnosis of urosepsis and commenced on IV temocillin and gentamicin. Despite treatment, his clinical condition did not improve, prompting a computed tomography (CT) scan two days after admission, which revealed a swollen right kidney with an ill-defined subcapsular haematoma consistent with WS (Figure 1). CT imaging demonstrated no evidence of renal neoplasm, angiomyolipoma, vascular malformation, or cystic disease, thereby excluding other recognised causes of WS and supporting anticoagulation with edoxaban as the most likely underlying aetiology.

**Figure 2:**
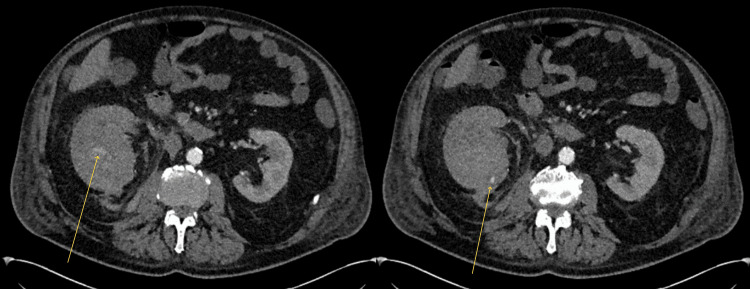
CT abdomen (axial view) in the arterial phase The CT images show dense contrast within the haematoma around the lower pole of the right kidney measuring 9.5 x 7 x 10 cm (arrows), suggestive of active bleeding. The bleeding is possibly from one or more subcapsular branches of one of the right renal arteries. CT, computed tomography

**Figure 3:**
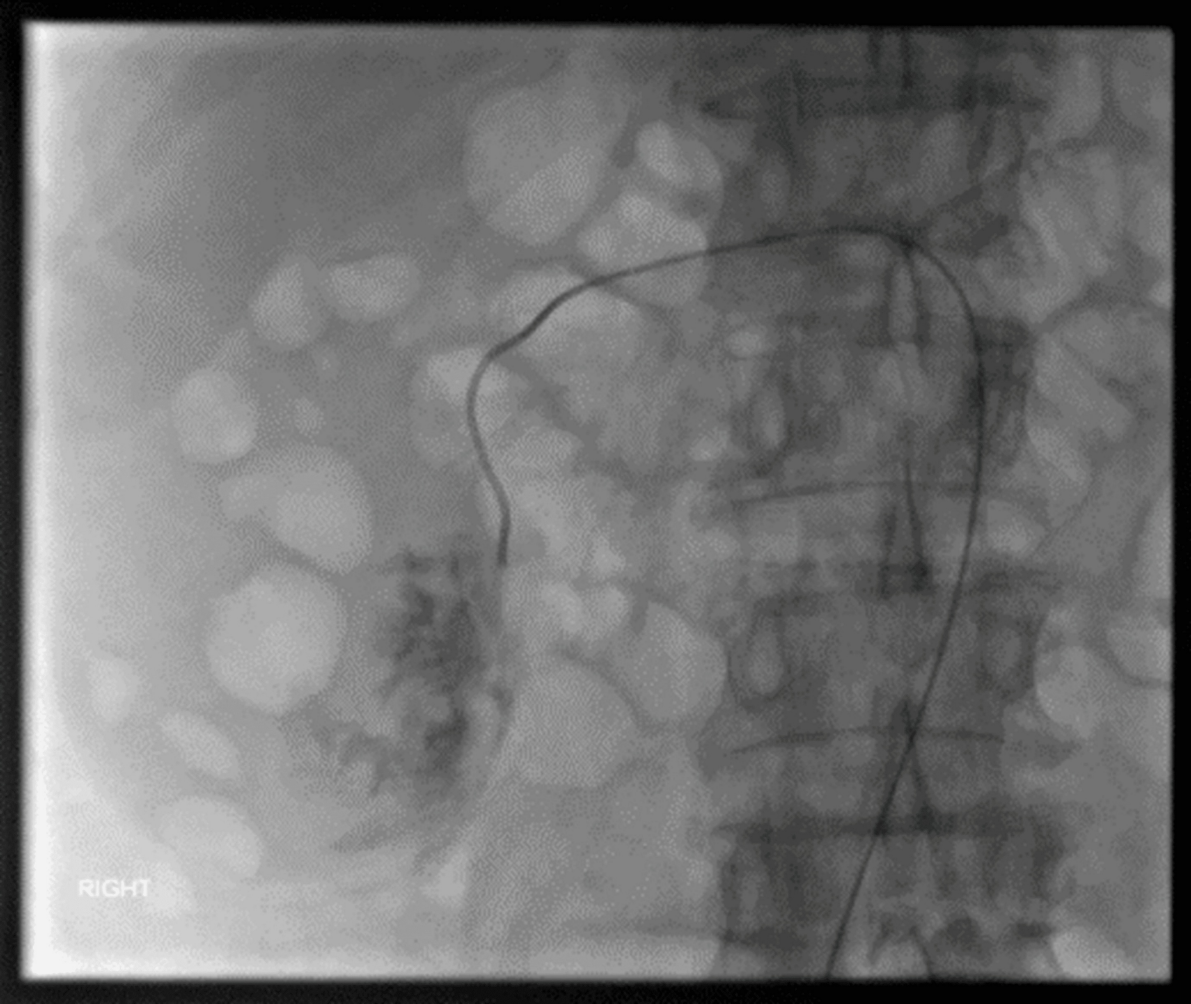
Fluoroscopic image during selective renal arterial embolisation There is brisk extravasation from a lower pole branch with injection of contrast, which was then embolised with a single 4 mm MReye® coil (Cook Medical, West Lafayette, IN, USA).

**Figure 4:**
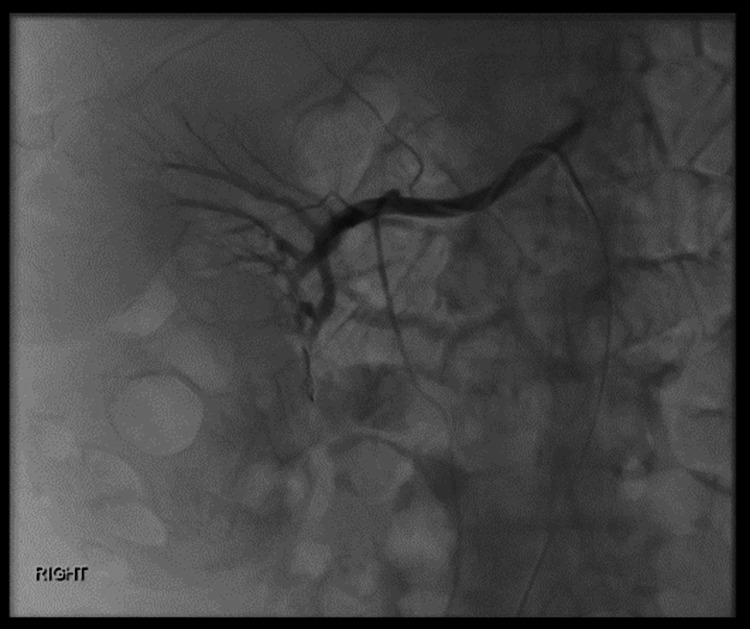
Selective renal angiogram There are at least five pseudo-aneurysms close to the renal capsule coming off different feeder branches, some of which cannot be clearly identified. These are not easily accessible for embolisation.

Concurrent blood results revealed a significant drop in haemoglobin to 76 g/L, prompting suspension of his edoxaban. A CT angiogram identified active bleeding at the lower pole of the right kidney, originating from subcapsular branches of the right renal artery (Figure 2). The patient subsequently underwent selective renal arterial embolisation. The procedure was partially successful, with embolisation of the major bleeders achieved, but some minor vessels could not be targeted (Figures 3-4). The patient’s international normalised ratio (INR) remained elevated at 1.5, compared to a previous level of 2.5 on admission, and the haematology team advised against specific reversal of edoxaban, given that more than 72 hours had elapsed since the last dose. Instead, intravenous vitamin K was administered to help correct the INR.

A follow-up CT angiogram performed one week later demonstrated persistent minor bleeding at the lower pole, though radiology deemed further embolisation of the entire kidney unjustified. Despite this, following three blood transfusions, the patient’s haemoglobin stabilised at 95 g/L, his inflammatory markers improved, and he was discharged after 18 days with outpatient follow-up arranged. Subsequent outpatient imaging included a dimercaptosuccinic acid (DMSA) renal scan, which demonstrated significantly reduced function in the right kidney (15%) compared to the left (85%). Laboratory tests also revealed a creatinine of 108 µmol/L, demonstrating an increase from his baseline of 87 µmol/L. Repeat CT imaging confirmed resolution of active bleeding but demonstrated a persistent subcapsular haematoma compressing the right renal parenchyma. Following review of the images and an assessment of the patient’s clinical condition, he was recommenced on edoxaban while being closely followed up by both the cardiology and the urology teams.

**Figure 5:**
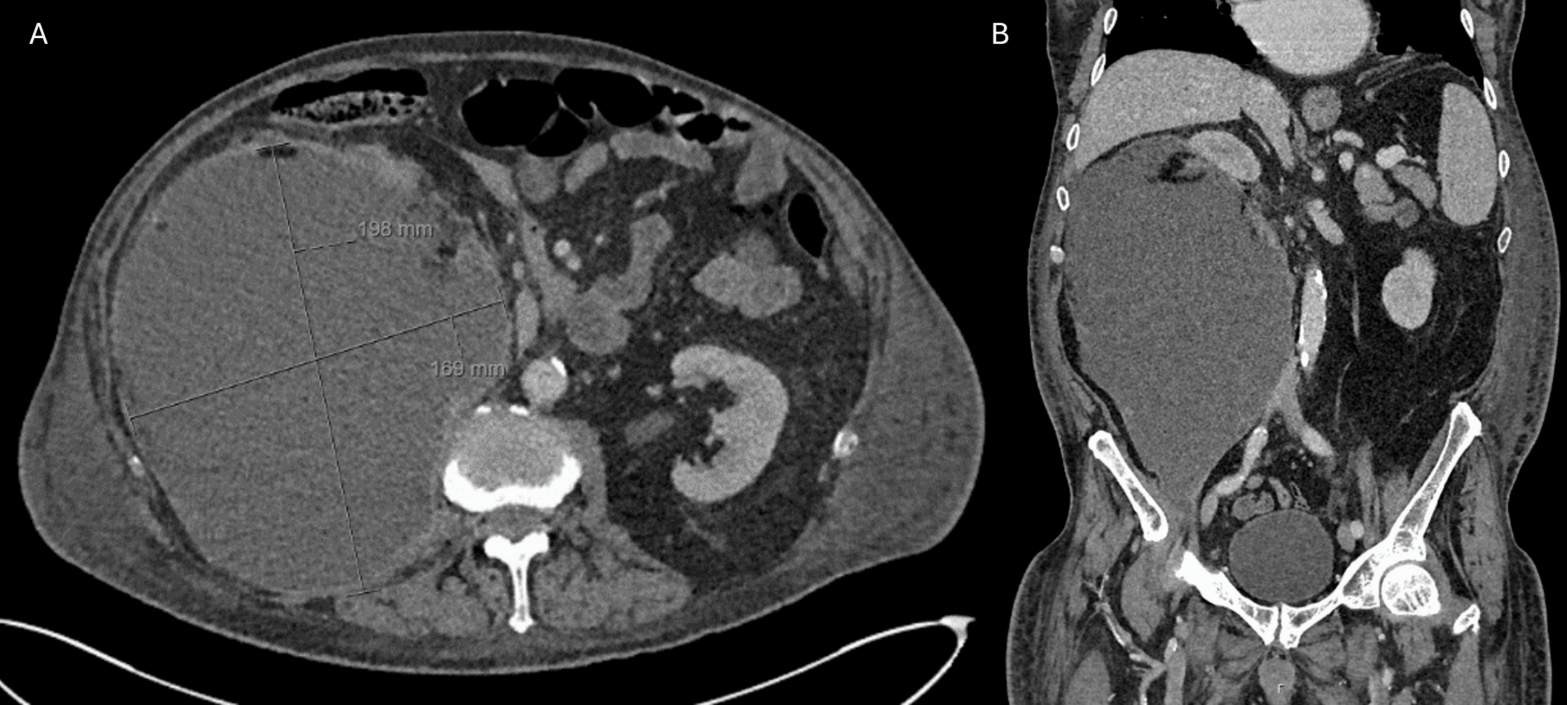
CT abdomen (axial view, A) and (coronal view, B) in the venous phase CT images demonstrate a large right-sided subcapsular/pararenal haematoma measuring 20 × 17 × 30 cm, exerting mass effect. The lesion contains extensive perinephric fat and multiple gas locules scattered throughout. CT, computed tomography

Approximately five months later, the patient was re-referred from the cardiology clinic to the Emergency Department with recurrent right flank pain and worsening lower limb oedema. Infective endocarditis was considered; however, multiple sets of blood cultures returned negative, and a urine culture revealed no significant growth. Given his renal history, a repeat CT scan was performed, which demonstrated a significant increase in the size of the right-sided subcapsular collection with features suggestive of superimposed infection (Figure 5). After discussion with the interventional radiology team, the findings were attributed to ongoing intermittent bleeding. His edoxaban was again withheld, and it was felt that definitive intervention was needed to allow the patient to safely resume anticoagulation.

Ultrasound-guided drainage of the collection was performed, followed by a second embolisation targeting residual bleeding. This resulted in a partial reduction in the size of the haematoma, now 8.5 × 6.2 cm in the axial plane. The admission was complicated by persistent hyponatraemia (serum sodium 121 mmol/L), which endocrinology attributed to syndrome of inappropriate antidiuretic hormone secretion (SIADH), likely secondary to the renal collection. With drainage, intravenous antibiotics and careful fluid management, the patient's clinical condition gradually improved, and the drain was removed several days later. Edoxaban was not resumed on discharge, and the patient continued to be monitored on an outpatient basis by both the cardiology and urology teams.

**Figure 6:**
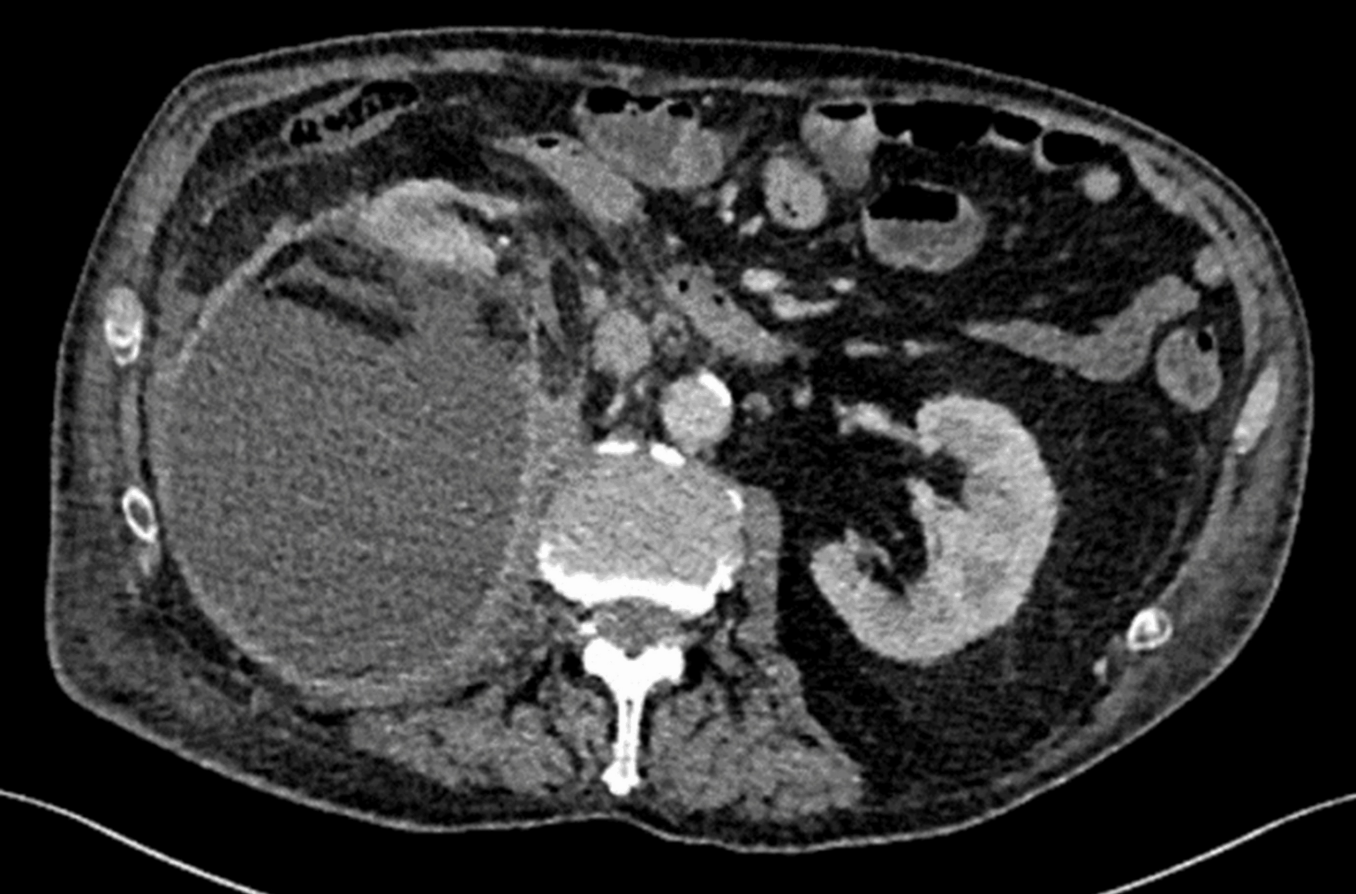
CT abdomen (axial view) in the portal venous phase CT demonstrates a significant increase in the size of the organised right perinephric collection, now measuring 24.5 × 13.0 × 11.4 cm, with concerns for superimposed infection. CT, computed tomography

Despite these interventions, the patient re-presented to the Emergency Department less than three weeks later with right flank discomfort secondary to a flank swelling, with persistent mucopurulent discharge ongoing for 48 hours. Imaging confirmed a recurrent increase in the size of the subcapsular collection, again with infective changes (Figure 6). The collection necessitated repeated drainage, and 1.8 L of pus were aspirated in total. A culture of the aspirate demonstrated growth of Group B Streptococcus and *Klebsiella pneumoniae*. A repeat CT showed successful reduction in the size of the collection. It also demonstrated a small functioning area in the right kidney, owing to some persistent perfusion. There appeared to be a possibility that some urine was being produced by the functioning nephrons in this area, which was draining into the perinephric collection and leading to infection/sepsis. The patient was subsequently discharged with a drain in situ, alongside follow-up imaging.

**Figure 7:**
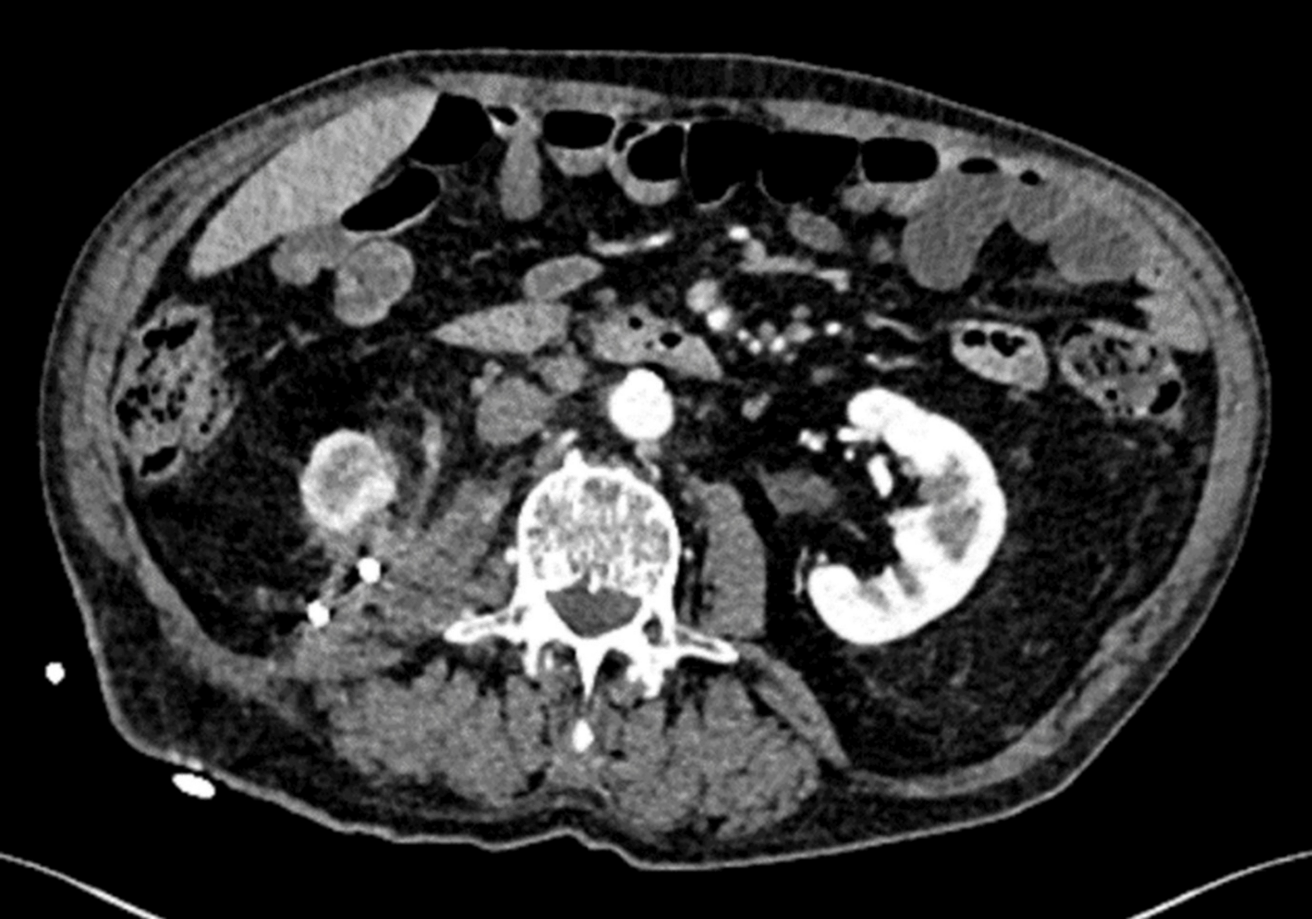
CT abdomen (axial view) in the portal venous phase CT demonstrates near complete resolution of the previous right retroperitoneal collection with trace residual perinephric fluid and stranding. The right-sided perinephric drain is visualised with the pigtail situated just posterior to the lower renal pole. CT, computed tomography

The latest CT scan (Figure 7), 12 months after the initial presentation, revealed near-complete resolution of the right retroperitoneal collection. Following both urology and radiology multidisciplinary team (MDT) discussions, and since the drain was only producing very minimal output, it was decided to remove the drain. The patient remains off edoxaban and has been discharged with ongoing follow-up.

## Discussion

This case highlights several critical challenges in diagnosing and managing WS in anticoagulated patients. The initial diagnostic delay occurred because the patient's symptoms mimicked a UTI, leading to missed early imaging. This is a pertinent issue, as patients often have a subtle presentation; literature indicates that Lenk’s triad is observed in approximately 20% of patients [3]. Given that anticoagulation increases the inherent risk of bleeding, a lower threshold for ordering a CT angiography scan in anticoagulated patients who present with flank pain or an unexplained drop in haemoglobin should be considered to evaluate WS. This offers both localisation of the bleeding source and identification of the underlying pathology.

Anticoagulation management posed another challenge. The decision to withhold or reverse edoxaban was complicated by the lack of clear guidelines. The elevated INR, despite direct oral anticoagulant (DOAC) cessation, suggests residual anticoagulant effects, underscoring the importance of early haematology consultation for reversal strategies. Moving forward, implementing a standardised protocol for restarting anticoagulation - potentially involving a delayed transition to a lower-dose DOAC or low molecular weight heparin (LMWH) - could mitigate recurrent bleeding risks. However, the decision to restart anticoagulation requires a careful risk-benefit assessment of thrombotic versus bleeding risks, taking into account patient-specific factors and emphasising shared decision-making between the patient and clinician.

Different types of anticoagulants work through various mechanisms in the coagulation cascade. Vitamin K antagonists, such as warfarin, function by inhibiting the production of vitamin K-dependent clotting factors [4]. Heparins, including unfractionated heparin (UFH) and LMWH, like enoxaparin, are another class of anticoagulants that act by enhancing the activity of antithrombin. DOACs include direct thrombin inhibitors (e.g., dabigatran) and factor Xa inhibitors (e.g., rivaroxaban, apixaban, and edoxaban), which directly target specific clotting factors [5].

With the increasing use of DOACs, case reports have begun to emerge that associate these newer agents, such as apixaban and rivaroxaban, with the occurrence of WS (Table 1). Some of these reports specifically highlight the clinical challenges in managing anticoagulation in patients who develop WS while taking DOACs, particularly concerning the optimal timing for restarting anticoagulation therapy to prevent subsequent thromboembolic events [6]. While DOACs are generally considered to have a safety profile that is comparable to, or even superior to, warfarin, spontaneous haemorrhages have still been reported with their use. DOACs are often perceived as having a lower bleeding risk than warfarin, although the potential for rare but severe haemorrhagic complications, like WS, does exist. Regarding edoxaban specifically, research suggests its use is associated with lower risks of major bleeding when compared with warfarin and certain DOACs (e.g., rivaroxaban and dabigatran) [7,8]. Compared to other DOACs, such as apixaban, edoxaban has been associated with a relatively higher risk of major and gastrointestinal bleeding, although it appears to carry a more favourable profile in terms of fatal bleeding risk [9]. Furthermore, managing WS in patients taking DOACs can be complex due to the shorter half-lives of these drugs and the varying availability of specific reversal agents [10]. This requires careful consideration of both the immediate bleeding risk and the subsequent risk of thromboembolic events.

**Table 1:** Wunderlich syndrome case reports attributed solely to anticoagulation

Reference	Patient Demographics	Anticoagulant Used	Clinical Presentation	Management Strategy	Outcome
Alshammari et al. (2025) [11]	47-year-old female with antiphospholipid syndrome	Warfarin (international normalised ratio, or INR 7.2)	Flank pain	Transarterial embolisation	Recovery
Mohammadian et al. (2023) [12]	71-year-old female with atrial fibrillation (AF) and metallic heart valve	Warfarin (INR > 6)	Flank pain	Nephrectomy	Recovery
Giovini et al. (2022) [13]	70-year-old male with AF	Warfarin (INR 2.5)	Sudden flank pain, anaemia	Conservative	Recovery
Chenna et al. (2018) [14]	74-year-old male with AF, previous stroke	Rivaroxaban	Sudden flank pain, hypovolemic shock	Conservative	Recovery
Maltês et al. (2023) [6]	79-year-old with AF and previous stroke	Apixaban	Severe flank pain, haemodynamic instability	Conservative	Recovery
Opancina et al. (2023) [15]	79-year-old female with COVID-19	Enoxaparin	Flank pain, hypovolemic shock	Conservative	Recovery

Existing case reports detail instances of WS occurring in patients receiving warfarin for various medical conditions, such as AF and mechanical heart valves [12]. Some of these reported cases involved patients with supratherapeutic INR levels, suggesting a potential dose-dependent relationship with the bleeding risk (Table 1). However, WS has also been reported in patients whose INR levels were within the established therapeutic range [13]. In some instances, patients who developed WS while on warfarin also had underlying renal conditions, such as cysts or tumours; the anticoagulation potentially exacerbated the bleeding from these pre-existing lesions.

In our specific case, incomplete initial embolisation resulted in recurrent bleeding, emphasising the need for thorough angiographic mapping of all bleeding vessels during the primary intervention. Early follow-up imaging (e.g., within 48 hours) could potentially identify residual bleeding and prevent subsequent complications. Additionally, in cases where embolisation fails to achieve complete haemostasis, earlier surgical consultation for partial or total nephrectomy should be considered.

Several case reports have linked heparin use, particularly during haemodialysis, to the development of WS, often in patients with a history of acquired cystic kidney disease (ACKD) [16]. It has been hypothesised that high-dose heparin boluses, administered during the dialysis procedure, might contribute to renal haemorrhage in these patients [17,18]. Enoxaparin, a commonly used LMWH, has also been implicated in case reports of WS, with at least one report suggesting its occurrence even at therapeutic dosage levels [15]. The association of heparin with WS in haemodialysis patients underscores the importance of carefully considering the bleeding risks in this population, especially given the high prevalence of ACKD among individuals undergoing long-term dialysis.

Based on the currently available literature, it is challenging to definitively determine which specific type of anticoagulant carries a higher risk of association with the occurrence of WS. This difficulty stems primarily from the fact that the majority of the evidence consists of individual case reports, alongside a notable lack of large-scale observational studies investigating this association. Warfarin has a longer history of clinical use compared to DOACs, which likely contributes to the greater number of case reports linking it to WS. The association between heparin and WS appears to be particularly prominent in the context of patients undergoing haemodialysis. While cases of WS associated with DOACs are increasingly reported, further research is needed to ascertain the true incidence and relative risk compared to traditional anticoagulants. Therefore, the current evidence is insufficient to establish a clear hierarchy of risk for WS among different types of anticoagulant classes.

The acute management of WS in anticoagulated patients demands a multidisciplinary approach. Immediate anticoagulant reversal is crucial; warfarin reversal typically involves vitamin K and prothrombin complex concentrate (PCC), whereas DOACs may necessitate specific reversal agents, such as idarucizumab for dabigatran or andexanet alfa for factor Xa inhibitors [2]. Heparin-induced bleeding is generally managed with protamine sulphate [19]. Concurrently, haemodynamic stabilisation with blood products and fluid resuscitation is critical to prevent hypovolaemic shock. In stable patients, conservative management with close monitoring may suffice. In cases where conservative management is insufficient, or if there is active bleeding, selective arterial embolisation may be employed to control haemorrhage while preserving renal function. This method boasts success rates exceeding 80% [20]. Surgical intervention, such as total nephrectomy or partial nephrectomy, is typically reserved for cases involving haemodynamic instability or failed embolisation.

For patients who have experienced WS while receiving anticoagulation, deciding when and how to restart anticoagulation therapy is complex. This decision requires careful evaluation of the indication for the anticoagulation, balanced against the risk of recurrent bleeding versus the potential risk of thromboembolic events if anticoagulation is withheld. Shared decision-making between patient and clinician is crucial, with clear communication of the risks helping to guide an individualised management strategy. A multidisciplinary approach, involving specialists from urology, interventional radiology, and haematology, is often essential in making these challenging management decisions.

## Conclusions

WS is a rare but potentially fatal condition that presents unique diagnostic and therapeutic challenges, particularly in patients receiving anticoagulation therapy. Clinicians need to maintain an index of suspicion in patients on anticoagulants who present with suggestive symptoms of the condition. In our case, cessation of edoxaban, multiple embolisations, drainage procedures, and close multidisciplinary follow-up underscored the importance of early suspicion, timely imaging, and coordinated management. Prompt diagnosis and a tailored management strategy, which includes cessation of anticoagulation and appropriate supportive or interventional treatments, are crucial for improving patient outcomes. Early cross-sectional imaging and multidisciplinary input are essential, particularly in cases where initial management fails to achieve haemostasis. Further research, including larger observational studies and the development of standardised management guidelines, is necessary to better understand the complex interplay between anticoagulation therapy and WS.
